# Progress in Medicine

**Published:** 1899-02-18

**Authors:** 


### PROGRESS IN MEDICINE.

### NEUROLOGY.

(*Continued from page* 331.)

**Epileptic Icterus.**—Féré[7] has recorded a case of epilepsy, in which each fit was followed by jaundice lasting about a week. The first urine voided after each attack contained bile pigments, and soon after the skin became yellow. The only accompanying symptom was a very slow pulse. Between the attacks the patient did not present the least symptom of hepatic disease, nor did she suffer from any marked gastric disturbance. Treatment by bromide prolonged the intervals between the fits, but did not modify the following jaundice. Jaundice has long been known to be a rare symptom in epilepsy, even Hippocrates speaks of bile as a cause of fits, but there are very few accurate reports of it in medical literature. The phenomenon is probably parallel with the jaundice resulting from intense emotion, of which many cases have been reported.

**Locomotor Ataxy.**—Redlich,[8] in a recent monograph on the pathology of locomotor ataxy, comes to the conclusion that the lesion of tabes has its origin in the posterior roots just at their point of union with the cord, and that this is anatomically a locus minoris resistentiæ. What the precise exciting cause of the change is, he is unable to determine. Actual meningitis is exceptional as a cause, nor do there appear to be primary changes in the ganglia of the posterior roots, or in the peripheral nerves. Syphilis was present in 65 per cent. of the men, and 23 per cent. of the women; and, in a few cases, other infectious toxic conditions seemed to have taken the place of syphilis as an etiological factor.

The treatment originally proposed by Fraenkel,[9] which consists in teaching the patient the co-ordination of muscles by carefully graduated exercises, has been found by numerous authorities to be the most successful as yet found. The following is a short description of the exercises. For the arms, the following directions are given: Sit in front of a table, place the hand upon it, then elevate each finger as far as possible; raising the hand slightly extend and then flex each finger and thumb as far as possible; do this first with the right and then with the left hand. Touch with the end of the thumb each finger tip separately and accurately, then touch the middle of each phalanx with the tip of the thumb. Sit at a table with a large piece of paper and a pencil, make a dot at each corner of the paper and one at the centre, and draw lines from the corner dots to the centre first with the right hand and then with the left. Put ten coins on the paper, pick them up and place them in a single pile, first with the right and then with the left hand. For the body and legs: Sit in a chair, rise slowly to erect posture without help of cane or arms of chair; then sit down slowly; stand with cane feet together, advance left foot and then return it; do the same with the right. Walk slowly ten steps forward and five back with help of cane. Stand without cane, but with the feet a little apart and the hands on the hips; in this position stoop down by flexing the knees and rise slowly. Stand without cane, but with the knees separated; raise the hands from the sides above the head; carry them downward and forward, and try to touch the toes. Walk along a fixed line on the floor by the help of a cane, placing each foot in turn on the line; then repeat without using the cane. Most of these exercises should be repeated several times, and the movements should be made with the eyes both open and shut.

Owing to disturbance of the sensory path tabetics have lost the sense of fatigue, so that there is some danger of overdoing the treatment. Two things are, therefore, insisted upon; every movement must be done with the greatest possible exactitude, and the seances, not more than two each day, must not last longer than eight or ten minutes.

In the pre-ataxic stage, the exercise treatment has in a number of reported cases prevented the development of inco-ordination, and in advanced cases remarkable results have been obtained; in a number of instances patients bedridden for three, four, and five years have been taught to walk without assistance. The improvement may last for years if the disease is stationary or only slowly progressive. The treatment is absolutely contra-indicated, according to Fraenkel, in cases of acute or subacute ataxia.

**Erythromelalgia.**—Collier[10] has recently published a series of cases of erythromelalgia with symptoms indicative of lesions of the central nervous system. The condition itself is one of acute vaso-dilatation over a definite area of the body associated with severe burning pain, or, if the part is analgesic, intense tingling. The effect of position on the affected region is remarkable, letting the foot (supposing this to be affected) hang down will bring on an attack, raising it causes the pain if it has occurred spontaneously to cease as long as elevation is kept up, and similarly the application of warmth increases, that of cold diminishes, the pain. The duration of an attack varies from half an hour to several weeks, but normally about three to four hours. The cases were: Six of disseminated sclerosis, two of tabes dorsalis, one of myelitis, and one of traumatic neurasthenia. In several cases there were at first only spontaneous attacks, afterwards these could be induced by the dependent posture, and later a condition of permanent vaso-motor palsy of greater or less degree came on. The accompanying sensory disturbances never preceded, but always followed or appeared with, the vascular storms. These sequences suggests an irritative lesion of nerve structures governing the blood-vessels as the cause of the phenomena.

**Polyneuritis of Pregnancy and the Puerperium** is discussed by Turney.[11] It must be distinguished from the local lesions of nerves, which owe their origin to the mechanical conditions of childbed or to the inflammatory conditions which succeed it, also from polyneuritis due to alcohol, lead and septicæmia occurring during pregnancy, or the puerperium. There are three clinical types of the disease—generalised neuritis, localised neuritis in either the upper or lower limbs, and neuritis affecting a single nerve. In the generalised type symptoms appeared at times varying from the fourth month of pregnancy to a fortnight after delivery. Pain came first and was followed by loss of muscular power, wasting and degenerative reaction, with tenderness of nerves. Mental symptoms were frequent, from mere emotionalism to acute mania. Recovery was complete in a few months in some cases, in others it was delayed for one or two years. In the other clinical types the local phenomena were very similar. The causation is probably to be explained on the auto-intoxication theory. The toxicity of the urine is known to be largely increased during pregnancy. This polyneuritis is probably only one of many phenomena due to auto-intoxication during pregnancy and the puerperium, viz., eclampsia, vomiting, acute yellow atrophy of the liver, recurring amblyopia, and various psychoses, including acute mania and melancholia. The alimentary canal is probably the birthplace of the poisons.

[7] Féré, Progrès Médical, Nov. , 1898. [8] Redlich, Der Pathologie der Tabischen Hinterstraugserkraukung. [9] Fraenkel, Deutsche Med. Wochenschrift. [10] Collier, Lancet, Aug. 13, 1898. [11] Turney, St. Thomas's Hospital Reports, Vol. XXV.

### RHEUMATISM AND GOUT.

**Bacteriology.**—Achalme's bacillus of acute rheumatism has been found by other investigators as well as himself. Some have discovered with it a diplococcus, and either organism appears to be able to set up endocarditis and other rheumatic symptoms. Riva, again, by the use of new cultivation methods obtained a different kind of microbe. Garrod[1] contrasts these results with those of Chvortek and Singer. The former failed to find any typical organism in the blood, urine, or synovia, and regards the disease as due to toxins elaborated in the tonsils and elsewhere. Singer met with pyogenic organisms in a large number of cases, and concludes that rheumatism is simply a form of pyæmia. However, against this view Garrod points out some forcible objections. Angus[2] failed to obtain any growths from the blood in the allied disease, Henoch's purpura, of which he had several cases with the classical symptoms of abdominal pain, hæmorrhage, ecchymoses, and at times nephritis. In true purpura the evidence of a specific organism is strong, one experimenter having given himself an attack by accidental inoculation. J. Mason, in New Zealand[3], has recently made cultures from the blood of a patient, and reproduced the disease in animals by inoculations from the cultures. An instance of the occurrence of the complaint in an infant may be noticed here, but it seems doubtful whether A. Madsen[4] has not mistaken a case of Henoch's disease for it. The child was 18 months old, and passed quantities of blood from the bowel, as well as showing numerous petechiæ. C. H. Lewis[5] found a pure cultivation of staphylococcus albus in the blood of a person who died from hæmorrhagic purpura, but he remarks that the blood here seems to be readily invaded by microbes, and if his case was not merely one of pyæmia with ecchymoses, the presence of the staphylococcus in the blood was perhaps a secondary phenomenon. The septic character of the gonorrhœal form of rheumatism is indirectly shown by the treatment employed by W. H. Marcy.[6] After all other treatment had failed he injected into the knee-joint sixty drops of a one in three thousand solution of perchloride of mercury with antiseptic precautions. A considerable improvement in the temperature and pain followed, and the injections were repeated twice a day for some time with complete success.

Complications.—D. B. Lees[7] points out that an acute dilatation of the heart is a common occurrence in acute rheumatism, even when other symptoms are slight, and endo- or peri-carditis absent; or it may intensify the symptoms of the latter conditions. This affection is possibly analogous to the dilatation seen sometimes in influenza, and is, like it, caused by the action of a toxin on the heart muscle. The evidence for the enlargement is based on tracings obtained by percussion, which show the remarkable increase in the area of dulness at an early stage of the attack, and a gradual reduction during recovery. In a second paper by B. D. Lees and F. J. Poynton it is reported that similar dilatation occurs in attacks of simple chorea in children, and out of 150 cases of fatal rheumatism in children dilatation was specially noticed at the post-mortem in no less than 92, or in 61 per cent. The heart sounds when dilatation comes on are altered, the first being weak and short, and the pulmonary second accentuated. The impulse becomes diffused and displaced to the left. This dilatation might be a useful aid to diagnosis in cases of arthritis of doubtful origin. Ewart suggests that many of these cases may be due to effusion, but the writers point to the absence of dulness to the right of the sternum, and the few (12) instances of effusion found in the 150 post-mortems.

F. J. Poynton[8] also records three cases of extensive venous thrombosis accompanying rheumatic carditis. In two instances both internal jugular veins were occluded, but in the third the left one only was affected. The writer professes himself unable to decide whether the cause of this complication is in the first place a thrombus or an inflammation of the vein. There was some evidence that partial thrombosis had existed for weeks before death, and the œdema of the face seen at the final stage of heart disease may sometimes depend on a similar condition. Such a state may be confused with the œdema of Bright's disease, with parotitis or angina. Similar thrombosis of the veins of the lower extremities has been recorded rather more frequently, but the condition is rare in any form.

Chronic hydrarthrosis of small joints has been brought forward by Galliard and Bernard as a special type of chronic rheumatism. Their patients had suffered from a subacute attack of true rheumatism previously, and the wrists and finger joints remained swollen and fluctuating without any redness of the skin or change in the bones or cartilages. The *Lancet*,[9] however, points out that in this and similar cases the occupation of the patients leads to a constant strain upon these joints. Where severe rheumatism has been present such strains are apt to cause local deformities, and the results must not be ascribed solely to any constitutional affection, but to local injuries in joints weakened by disease. The diagnosis of muscular rheumatism is also difficult at times. Thus A. G. Miller[10] notices that it may be mistaken for articular affections when the muscle overlies a joint, but there is little swelling in myositis. He thinks that a crackling may sometimes be heard in these cases from effusion into the subcutaneous tissue. Besides ordinary remedies he recommends a few drops of actea racemosa. An apparent anchylosis of a joint may be due to the stiffening of a muscle from chronic myositis.

Treatment.—Chauvet, of Royat,[11] speaks very highly of the electric bath in chronic rheumatism, and claims that it is more rapid in its results than any other treatment which he has tried. From fifteen to thirty milliampères are employed, and the electrodes consist of charcoal covered with flannel. The cases where Heberden's nodes were present, however, showed no improvement. Superheated air is now very largely used, and by it the Abstractor of these records has restored complete mobility to the anchylosed hands of one patient who suffered from typical rheumatoid arthritis. The apparatus used was Tallerman's. This, however, is a costly one, and F. Krause[12] improvised one for himself, with which he obtained valuable results in many cases both of rheumatism, rheumatoid arthritis, chronic gonorrhœal arthritis, chronic effusions, and periostitis. This author commenced with a temperature below 90 deg. C., and gradually increased it up to 100 or 135 deg. C. He used the oven twice a day for an hour or more, taking care to provide free exit for the moisture, and indeed for the expanding air. The anodyne effects are very marked, but cures must not be expected in every case. Great care must be taken to avoid a general chill after the local bath. A. Bier[13] acknowledges the remarkable reduction of pain and stiffness brought about by this treatment. This is produced by the active hyperæmia caused by it, but he claims that better results are obtained by passive hyperæmia, and that the method is simpler. The leg, for instance, is bandaged from the toes up to the affected knee, and above the knee an elastic bandage is applied over another bandage. Each case must be watched, lest the limits of safe pressure be exceeded. Pain must not be caused, and the bandage should relieve any pain previously present. The bandage may be employed during either day or night, but where the joint shows much pain it may be used continuously, but changed every twelve hours. The joint itself must not be bandaged.

Linossier and Lannois[14] return to the application of methyl salicylate, which is very rapidly absorbed through the skin when properly employed, without causing stomach irritation or tinnitus in ordinary cases. In acute rheumatism the treatment may be given twice a day. No excipient should be employed, but one, two, or even three drachms should be painted on the skin and carefully covered by several layers of indiarubber firmly applied. They do not recommend the vegetable oil of winter green.

Harlet[15] recommends the rectal injection of salicylate of soda in a cup of bran water, with a little opium if necessary. In acute cases he gives two injections each of one drachm or a drachm and a half, reducing this by 15 grains every second day if improvement is manifest. The drug is completely absorbed in six or eight hours, and produces no cardiac or gastric complications.

Levison,[16] in an elaborate paper on the diagnosis of gout from rheumatoid arthritis, where he recognises the possibly infectious origin of the latter disorder, records that he found some cases of this disease benefited by electrical treatment or electrolysis, but that warmth in a hot air bath with a temperature up to 140 deg. Fah., seemed to give the best results. He at length devised a local hot air bath of the type now in use in this country, and reports highly valuable results.

Some cases, indeed, of old standing cannot be cured, but the hot air relieves the pain, and icthyol may be given for the improvement of the general health. In one of his patients stiffness and pain in the knee-joints of six years' standing disappeared after 26 days' treatment by hot air and massage, and in another severe and increasing pain and stiffness of a year or two's duration was absolutely cured, the patient having perfect use of his arms six months after the treatment was left off.

[1] Practitioner, Sept. [2] Lancet, June 4. [3] Australasian Med. Gaz., May 20. [4] Brit. Med. Jour., June 15. [5] Med. Rec., May 28. [6] Med. Rec., July 2. [7] Lancet, July 4. [8] Lancet, July 11. [9] Lancet, June 25. [10] Lancet, June 18. [11] Brit. Med. Jour., July 23. [12] Brit. Med. Jour., June 25. [13] München Med. Woch, Aug. 2. [14] Brit. Med. Jour., June 25. [15] Clinical Jour., July 4. [16] Practitioner, Sept.

### THE ALIMENTARY TRACT.

**Functions of the Stomach.**—Cannon[1] has investigated gastric digestion and gastric movements by feeding cats with food mixed with subnitrate of bismuth, and then following the changes in the contour of the stomach by means of the Röntgen rays. The stomach may be divided into three parts—the cardiac end, or fundus, extending from the left edge to half-way across the organ; the antrum, which is just at the beginning of the small intestine; and the preantral portion, extending from the middle nearly to the pyloric opening. In the fundus the softening and maceration of the food, and the salivary digestion of the starches proceeds for some time without the admixture of the acid gastric juice. The peristaltic waves then force the food into the preantral space, which has now become a narrow tube. Here it oscillates backwards and forwards for a time, so as to be thoroughly mixed with the gastric juice, and, finally, when sufficiently softened, passes through the pylorus. The pylorus only opens at irregular intervals, and the arrival of a hard morsel causes the sphincter to open less frequently. The fact that the fundus is largely engaged in completing salivary digestion explains the well-known fact that such drugs as taka-diastase and pancreatin carry on their digestive action in the stomach, which should presumably be interfered with by the acid gastric juice. Marked nervous excitement has an inhibitory effect upon the movements of the stomach.

Carvallo and Pachon[2] have successfully removed the entire stomach from a cat with a view to showing that the chemical, peptic, and antiseptic functions can be vicariously discharged by other parts of the alimentary apparatus. The animal was able to digest proteids, starches, and fats if cooked, and three and a half months after the operation had increased in weight. Lack of appetite pointed to the stomach playing an important part as a peripheral sensory organ.

Schlatter[3] gives further observations of his well-known case of total extirpation of the stomach in the human subject. He mentions that a second successful operation has now been performed by Brigham, of San Francisco. It is now nearly nine months since Schlatter operated on his patient. There has been no return of the malignant growth, and the patient's weight has increased by 18½ pounds. The diet now differs very little from that of other patients in the hospital, but there is a constant feeling of pressure in the epigastric and hypochondriac regions after a hearty meal. The remarkably small quantities of chloride of sodium in the urine have been regarded by some observers as evidence in favour of Koppe's view that the hydrochloric acid of the stomach is formed from the chlorides of the food on the surface of the gastric mucous membrane. Hoffmann, however, maintains that the stock of chloride of sodium essential for the organism became exhausted during the preceding long period of partial starvation, and has not yet been made up to its full amount. The acidity of the urine is high, and the fall in the acidity which occurs normally after a hearty meal is entirely absent in this case. The absence of gastric juice seemingly makes no difference to the assimilation of albumin, and a steady increase of the circulating albumin is taking place. The digestion of fat is normal, and there is no sign of putrefaction of the intestinal contents. As in the normal individual, the elimination of nitrogen undergoes an increase two hours after meals, and the removal of the stomach has not caused the excretion curve to deviate in any way from those which are obtained under normal conditions. Examination of the gastric contents[4] should be resorted to in all chronic affections of the stomach with doubtful diagnosis. Thus it is useful in chronic gastric catarrh, achylia gastrica, incipient cancer of the stomach, hyperchlorhydria, gastrosuccorrhœa, erosions of the stomach, and ischochymia. If vomiting is present the use of the tube is unnecessary. The results should be combined with all other data of the case. In acute cases, and in cases of ulcer with hæmorrhage, it is best to forego the examination. Free hydrochloric acid in the stomach contents may be detected by adding to the filtered material a few grains of detrose. The fluid is then poured out on a clean porcelain plate and a few drops of solution of alphanaphthol added. After gently heating a violet-blue colour is developed, which rapidly changes to black.

**Gastric Hyperacidity.** — Joslin[5] recommends the strengthening of the abdominal muscles by exercise or massage, and the administration of tincture of nux vomica in doses reaching sixty to ninety drops per diem. Should these measures not suffice the stomach tube should be employed. Thirst is best met by rectal saline injections. The proteid diet certainly suits mild cases best, and if carbohydrates are given, foods in which the starch has been dextrinised are indicated, or diastase administered. Proteids may relieve the pain accompanying this disorder, and this is better than resorting to diluents. Food should be in small bulk. All alkalis are of temporary value. The diet should include plenty of fat. Dubard, in the Journal of the American Medical Association, states that acidity and pyrosis are intensified by sodium bicarbonate if the condition be due to the presence of organic acids, but relieved if the disease be true hyperacidity. Olivette[6] finds the symptoms of hyperacidity relieved by introducing into the stomach through a tube 150 to 230 grains of subnitrate of bismuth suspended in water. This is done each day before breakfast for three weeks. The bismuth forms a protective coating over the gastric mucous membrane, and in no way lessens the quantity of gastric fluid, or the movements of the stomach.

**Dilatation of the Stomach.**—Maynard[7] in 87 cases of chronic catarrh of the stomach found chronic dilatation in 19, and regards the catarrh as a causative agent. Nausea, interscapular pain, and a sense of fulness and weight at the lower end of the sternum, were the commonest symptoms. The administration of a dry finely divided diet and the strict limitation of fluid are essential to successful treatment. Of drugs, hydrochloric acid and strychnine were the most useful.

Von Jürgensen[8] reports a case of severe cerebral symptoms and death from excessive dilatation of the stomach. The disease was of ten years' duration, and proved at the post-mortem to be due to cicatrisation of pylorus and duodenum. The patient, a man of 43 years, suffered from anorexia, vomiting, great weakness, and severe thirst. The signs of dilated stomach were marked. Suddenly the intellect became slightly clouded, atactic movements and general spasms commenced, the urine was suppressed, and thirst and vomiting increased. Coma, spasms, cyanosis, and loss of reflexes lasted for two days, when the patient died. As diabetes, cancer, and cholæmia could be excluded, death was due to an intoxication of some kind, probably uræmic ; another factor largely responsible for the symptoms being the lack of water in the blood and tissues, due to the lessened absorption from the altered mucous lining of the stomach. A very uncommon cause of dilatation of the stomach is congenital hypertrophic stenosis of the pylorus. Including the case described by Cautley,[9] 20 such cases are now on record. The disease is due to hypertrophy of the unstriped circular muscle fibres forming the middle coat of the pylorus. The symptoms which generally arise during the first month of life are vomiting persistently and without cause, absence of bile from the vomit, constipation, marasmus, pyloric tumour, dilatation of stomach, and the absence of signs of gastritis. Surgical measures offer the best prospect of cure.

**Gastric Tetany.**—Trevelyan[10] describes two cases of tetany complicating gastric dilatation. The first was in a woman of 45 years, in whom all the symptoms and physical signs of dilated stomach were pronounced. There was wasting and signs of a pyloric tumour. The urine contained a trace of albumin. On the third day after admission there was persistent vomiting, and tetany supervened. The spasms affected the hands, arms, feet, and muscles of the jaw. The temperature fell to 94·6 deg., with signs of collapse. Death was preceded by a more generalised spasm. Post-mortem, carcinoma of the pylorus was found. In the second case—also a woman—the gastric dilatation was due to the fixing of the pylorus by adhesions. Tetany set in a day after admission, and was fatal in a few hours. The chief factor in the causation of gastric tetany would appear to be auto-intoxication from fermentative changes taking place in the partially-digested food in the stomach. The theories of reflex neurosis and of insufficient absorption of fluid are fast becoming obsolete. Though often associated with renal disease there is no evidence to show that tetany may be the result of uræmia. Probably the kidney change is excited by the excretion of the toxins. Such toxins have been isolated, and when injected into dogs excite convulsions. Seventy-five per cent. of cases of gastric tetany are fatal. Treatment by lavage of the stomach with a soft tube is most effectual, and fluid should be supplied to the body by means of rectal injections.

McKendrick's case[11] was that of a man aged 26, who for four days before admission had been seized with cramps and tingling pains in both extremities, with spasms, vomiting, and some collapse. On admission the tetanic spasms occurred every few hours, the muscles of hands and fingers being in a state of semi-contracture in the intervals. There was repeated vomiting and some albumin in the urine, no casts, no sign of dilated stomach. He died on the fifth day from exhaustion, and at the post mortem marked gastric dilatation and cicatricial contraction of the pylorus were found. The premonitory symptoms in this case were tingling sensations, chills, drowsiness, swelling of the head and tongue. There is no proof that debility from exhaustion causes hyper-excitability of motor cells, and consequent tetany. It is more probable that toxins formed by fermentative processes are the causative agents.

[1] Amer. Jour. of Physiology, May, 1898. [2] Lancet, Aug. 20, 1898, p. 497. [3] Lancet, Nov. 19, 1898, p. 1,314. [4] Med. Rec., July 9, 1898. [5] Boston Med. and Surg. Jour., No. 17, 1898. [6] Therap. Monatshafte, 1898, Heft. 4, S. 181. [7] Brit. Med. Jour., Oct. 15, 1898. [8] Amer. Jour. Med. Sci., Sept., 1898. [9] Brit. Med. Jour., Nov. 12, 1898. [10] Lancet, Sept. 24, 1898. [11] Ditto.

